# The development and evaluation of an in-vitro shoulder simulator with active muscle simulation

**DOI:** 10.1038/s41598-023-31200-y

**Published:** 2023-03-17

**Authors:** Ruipeng Guo, Manuel Ferle, Dennis Nebel, Christof Hurschler

**Affiliations:** 1 Laboratory for Biomechanics and Biomaterials, Department of Orthopaedic Surgery - DIAKOVERE Annastift, Hannover Medical School, Anna-Von-Borries-Str. 1-7, 30625 Hannover, Germany; 2 Department of Orthopedics, the First Affiliated Hospital of Anhui Medical University, 218 Jixi Road, Hefei, 230000 China; 3 Chair of Ergonomics, Technical University of Munich, Boltzmannstr. 15, 85748 Garching b. München, Germany

**Keywords:** Biomedical engineering, Hardware and infrastructure, Experimental models of disease, Preclinical research

## Abstract

The purpose of the present study was to develop a novel active in-vitro shoulder simulator to emulate all forms of planar and non-planar glenohumeral motions with active muscle simulation on cadaver specimens or shoulder models and to critically evaluate its performance. A physiologic shoulder simulator, driven using simulated muscle force, was developed to dynamically realize accurate kinematic control in all three rotational degrees of freedom (DOF) under physiological kinetic boundaries. The control algorithm of the simulator was implemented using three parallel running independent control loops, which regulate the forces of individual muscles in the respect DOF and work asynchronously in disparate sequences adapted to specific motions (abduction, flexion/extension and rotation). Three cadaveric specimens were used to evaluate the kinematic and kinetic performance of the simulator during simulated motions. High kinematic accuracy (maximum mean deviation ≤ 2.35° and RMSE 1.13°) and repeatability (maximum and average SD of ≤ 1.21° and 0.67°) were observed in all three rotational DOF investigated. The reliabilities of all individual muscle forces actuated in the simulator during planar and non-planar motions were generally excellent, with the 95% CIs of ICC estimates of > 0.90 for most instances (30/36). A novel shoulder simulator with active muscle simulation was developed and evaluated. Its capability to reproduce kinematics and kinetics in a physiological range for all DOF was systematically evaluated for multiple kinetic and kinematic outcome variables. The presented simulator is a powerful tool for investigating the biomechanics of physiological and pathological shoulder joints and to evaluate various surgical interventions. Acquisition of reliable data in joint kinetics and translational kinematics during active motions is critical to assess shoulder pathologies and appropriate treatments. We provide a unique muscle activated physiologic shoulder simulator, which allows the comprehensive acquisition of joint kinematic and kinetic data during repeated realistic planar and non-planar motions.

## Introduction

In comparison to the hip, the other large human ball and socket joint, the glenohumeral joint is characterized by a unique geometry with limited congruency and a relatively unconstrained articulation. This allows for a large range of motion, whereby stability is provided by the passive stabilizers of capsular and ligamentous structures as well as the active stabilizers of the rotator cuff and deltoid muscle groups^1^ In part due to its distinctive anatomical structure and active stabilization, the shoulder is susceptible to injury, including instability, rotator cuff tear, labrum tear and capsular ligament sprain^2–4^ Thus, when investigating shoulder pathologies and their treatment experimentally by means of biomechanical simulators, the representation of the anatomy and the function of the active and passive stabilizers is essential.

Acquisition of reliable data in joint kinetics and translational kinematics, especially during non-planar motion, is difficult if not impossible in vivo, but nonetheless critical to assess the functional effects of shoulder pathologies and their proposed or applied treatments. Experimental (in vitro) biomechanical testing can supplement knowledge from gained clinical tests such as the Lift-Off test and hyper extension-internal rotation (HERI) tests for diagnosing anterior/inferior instability, kinetic analysis for investigating the effects of massive rotator cuff tears, and the functional tests to evaluate reverse total shoulder arthroplasty^5–7^ Thus, notwithstanding that studying shoulder biomechanics in vivo is generally the most important source of information, difficulties in directly obtaining kinematic and other data are a serious limitation. In vitro cadaver models offer the advantage of applying invasive tracking and measurement methods and of alteration and manipulation of the joint with the advantage retaining much of the native anatomy. Thus several passive and active shoulder simulators have been built in the recent decades with the goal of simulating physiologic and pathologic kinematics and identifying the significant contributors^8–12^ The use of the passive shoulder simulators without active muscle simulation is largely limited to research questions associated with the stabilizing soft tissues of the joint. Nonetheless, previous studies have illustrated the importance of the musculature in creating and maintaining glenohumeral stability during active joint motion. For this reason, active shoulder simulators with the aim of representing the periarticular muscles have been designed with the aim to realistically reproduce dynamic joint motion^13,14^ An early representative model of a muscle-driven simulator was developed by Wuelker et al. in 1995, which realized reliable and dynamic shoulder abduction by muscle actuation through hydraulic cylinders, to which the rotator cuff and deltoid muscles were connected via steel cables with additional force sensors suspended in between. In addition, ultrasonic sensors were used to record arm kinematics^10,15^ Even though it represented a great advancement over other simulators in that era, simulated motion was limited to abduction and muscle forces were increased linearly with constant ratios of relative activation which does not account for the strong nonlinear behavior of the musculature. Active motion in all three rotational degrees of freedom (DOF) (e.g. active abduction, flexion and rotation) was first achieved in a refined simulator with real-time kinematic feedback and closed loop kinetic control by Giles et al^16^ However, its capability was limited to performing secondary DOF motions (e.g., plane of elevation and axial rotation) at small angles of abduction (< 15°) and complex non-planar motions in multiple DOF. In contrast to Wuelker et al., the working groups around Kedgley and Giles used low-friction pneumatic actuators controlled by compressed air via proportional pressure controllers. An electromagnetic and an optical tracking system, respectively, were used to record the kinematics. Thus, despite some notable advances, to date no in vitro shoulder model is available which allows for comprehensive emulation of joint kinematics and kinetics during repeated planar and non-planar motions.

Therefore, the aim of this study was to develop a novel active shoulder simulator with physiologic range muscle actuation and to critically evaluate its performance under repeated planar and non-planar motions. We hypothesized that the simulator would provide accurate and reliable kinematic and kinetic data during controlled motions in multiple DOF.

## Materials and methods

### IRB approval

The experiments were performed on human shoulder specimens obtained through a body donor program (Science Care Inc., Phoenix, AZ, USA), informed consent was obtained by Science Care from all tissue donors and/or their legal guardian(s). All procedures were performed in accordance with the ethical standards of the institutional research committee and with the 1964 Helsinki Declaration and its later amendments, or with comparable ethical standards, and institutional IRB approval was obtained. All the experimental protocol/s was/were approved by a named institutional and/or licensing committee (Ethics Committee of the Hannover Medical School, No. 3005–2016).

### Simulator setup

**Figure 1:**
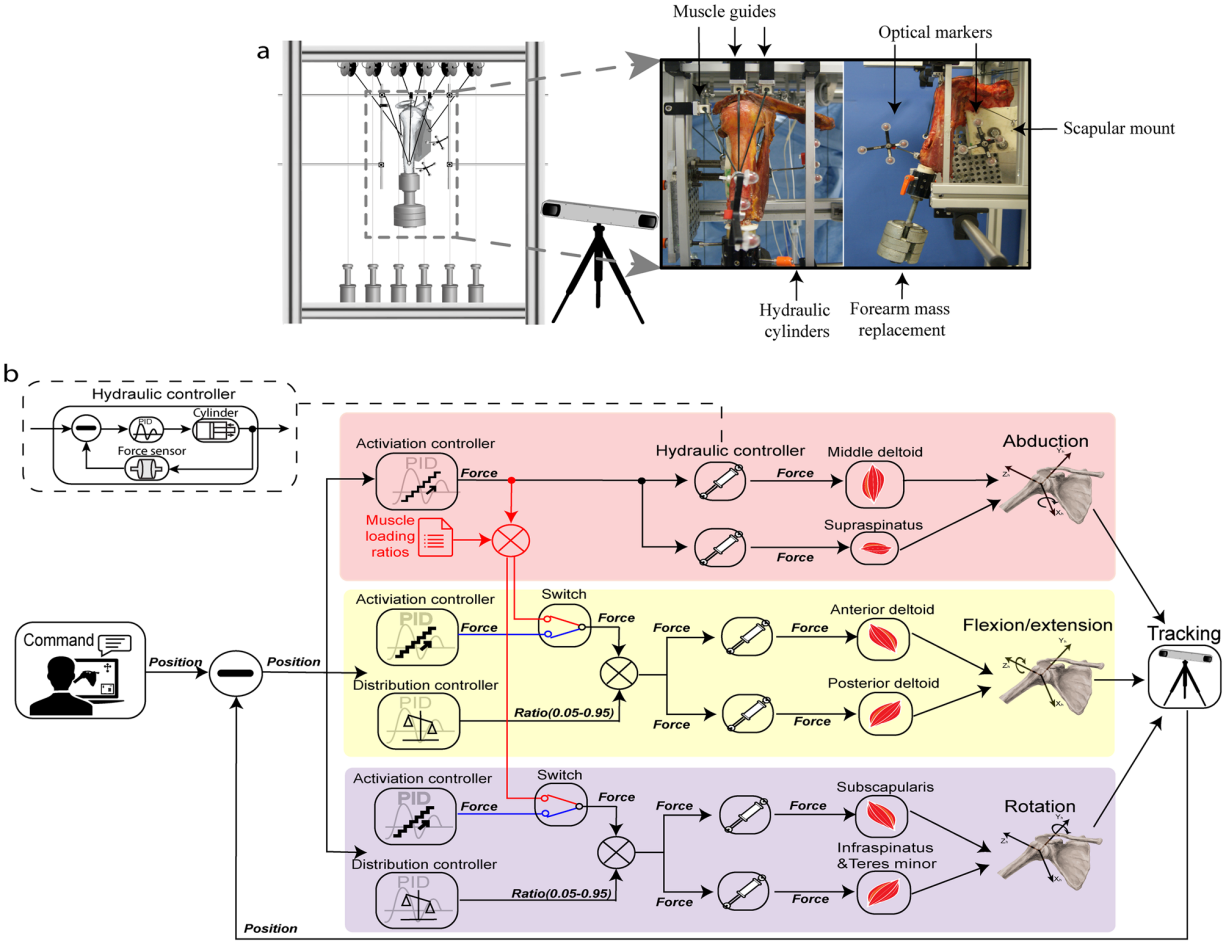
Setup of the active shoulder simulator. (**a**) The specimen is mounted on the assembled testing frame and six low friction cables and muscle guides was customized to represent the lines of action of individual muscles. The optical motion tracking system is consisted of the camera and optical markers on the humerus and scapula. (**b**) Control algorithm in the simulator. The colored boxes denote the data flow of loop specific feedback and three independent control loops regulate the forces of individual muscles in respect DOF. The dashed black boxes are the loop of force control in the hydraulic cylinders. There are two switches of operating sequence in the flexion/extension and rotation loops: (1). Cascade control (red gear): during active abduction, summation forces of the muscle couples in the secondary DOF (anterior&posterior deltoid and subscapularis&infraspinatus/teres minor) are commanded by the product of middle deltoid force and prior loading ratio; (2). Parallel control (blue gear): during active flexion/extension and rotation, summation forces of the muscle couples are determined by the activation controllers.

The novel shoulder rig consists of a mounting frame, allowing the fixation of the scapula, a robust cable-guiding frame (65 × 40 cm) for muscle guide placement and a hydraulic actuation unit consisting of six low friction hydraulic cylinders with hydraulic pump and hardware controller (Parker, USA), allowing force application to muscle wires (Fig. 1a). Furthermore, the system consists of six single-axis miniature load cells (Model Nr. 8417–6005, 5 kN, Burster gmbh&co, Germany), each connected between a muscle cable and a hydraulic cylinder. In total six muscle wires were used to simulate the rotator cuff and the deltoid of the shoulder.

For testing a cadaveric shoulder joint consisting of the scapular bone, the proximal part of the humerus and the soft tissue capsule of the glenohumeral joint was integrated into the test rig: The humerus was allowed to hang freely without constraining any DOF, and a rod was cemented to the distal humerus for attachment of a mass (3.2% body weight) to simulate the weight and center of mass of the missing forearm^15,17^ Two customized ceramic low friction muscle guides were placed at the supero-lateral rim of the acromion and one guide at the distal rim of the clavicle to direct the line of action of the three heads of the deltoid muscle: the anterior (AD), middle (MD) and posterior (PD) deltoid. Two further guides were placed at the scapular centroid of subscapularis (SSC) and infraspinatus & teres minor (ISP/TM) along the lateral border of the scapular. A further low friction pulley was used to guide the line of action of the supraspinatus (SSP) in the fossa supraspinata.

An optical tracking system (NDI Polaris P4, Northern Digital Inc., Waterloo, Canada) was used to record the kinematic data of the humerus relative to the scapula, with two passive clusters of four retro-reflective markers each, firmly attached to humerus and scapula, respectively.

### Coordinate systems

**Figure 2:**
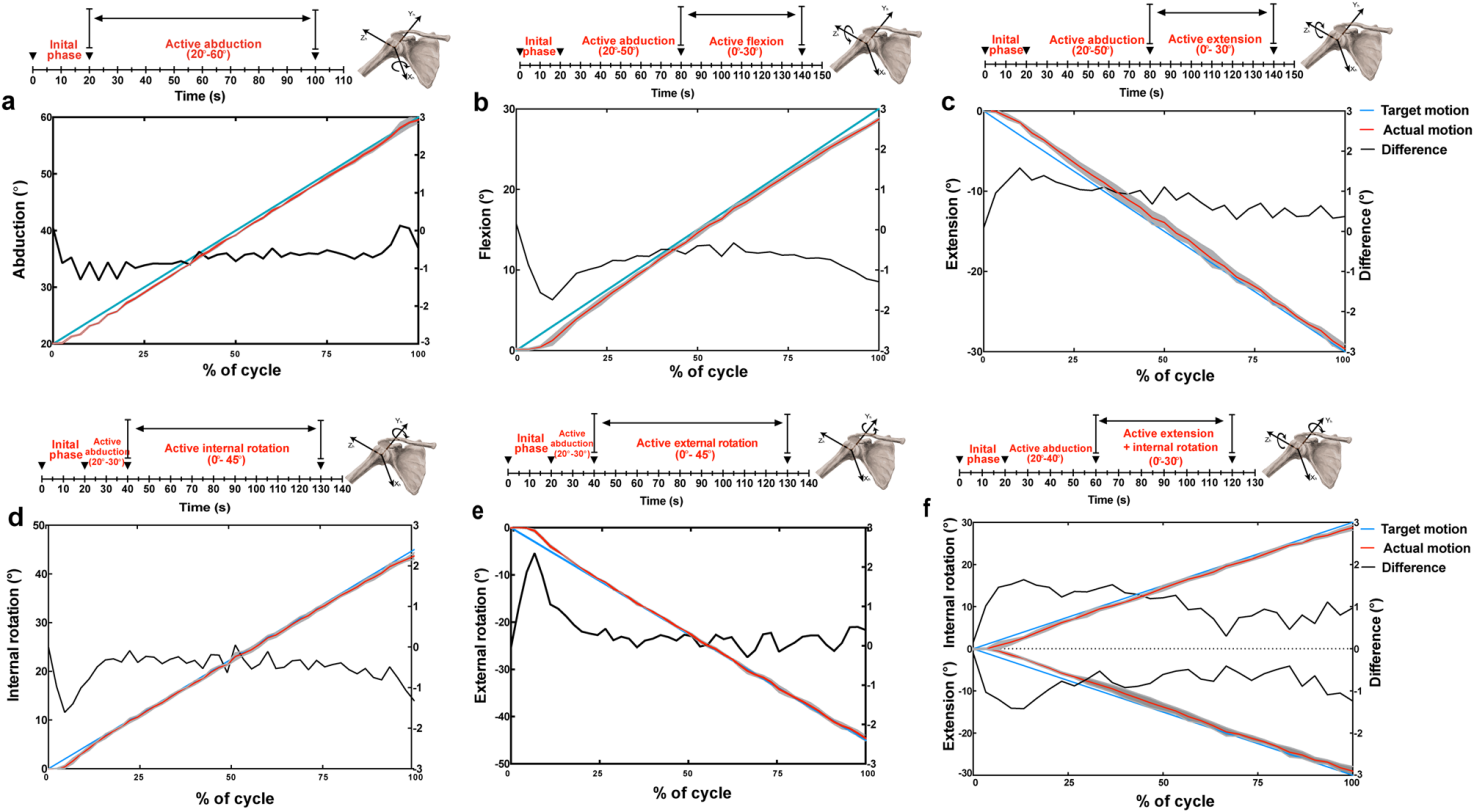
The performance of the simulator during active motions. (**a**–**f**)**.** The blue lines represent the target motions for active simulation and the red lines indicate the average of actual motions of all specimens during active abduction (**a**), flexion (**b**), extension (**c**), internal rotation (**d**), external rotation (**e**) and extension&internal rotation (**f**) cycles; the grey areas represent the standard deviations of actual motions; the black lines represent the mean deviations between the target and actual motions.

Local anatomical coordinate systems of the humerus and the scapula were defined according to the recommendations of the International Society of Biomechanics (ISB) to describe glenohumeral joint motion^18^ As part of the setup procedure, the kinematic center of rotation of the humeral head was defined as the point moving least when manually passively rotating the joint in three DOF while applying a horizontal centering force to the glenohumeral joint^19^ The humeral coordinate system was then placed at the thus defined center of rotation of the humerus. The origin of the scapula coordinate system was placed at the anatomical landmark angulus acromialis (AA). To avoid gimbal lock during the initiation of abduction, the Cardan rotation sequence *XZY* was used to represent glenohumeral position, instead of the ISB-recommended Euler *YXY* rotation sequence^20^ Internal/external rotation (IR/ER) was defined as the rotation around humerus shaft axis (Y-axis), flexion/extension (F/E) as the rotation around Z-axis of the humerus being created by the two anatomical landmarks on the medial and lateral epicondyles of the humerus showing lateral, and abduction as the rotation around the axis being perpendicular to the other two axis (X-axis) (Fig. 2). Anterior/posterior (AP), superior/inferior (SI) and medial/lateral (ML) translations were defined as displacements of the center of rotation of the humeral head with respect to the scapular coordinate system.

### Control algorithm

The control algorithm of the shoulder simulator was adapted from Giles et al. and implemented as a dedicated program in LabVIEW (version2017, National Instruments, USA), with which kinematic inputs are translated into glenohumeral motion. In this controller, the input rotational kinematics are the setpoints (SP), the muscle forces are the control variables (CV), and rotational kinematics of the glenohumeral joint as measured by the motion tracking system are the process variables (PV). Muscle forces were generated using hydraulic actuators. The muscle forces actuating the three rotational DOF were governed by three separate and independent control-loops, which drive the “prime mover” muscles in their respective DOF (Fig. 1b). Each of the loops consist of individual PID force controller for abduction, flexion/extension, and internal–external rotation respectively, and will be described in detail below.

In order to implement a closed-loop muscle activation, a combined set of loading ratios calculated from physiological cross sectional area (pCSA) and electromyographic (EMG) data of the simulated muscles from the literature were initially loaded (Table 1)^14^ Subsequently, individual muscle forces were automatically tuned by the control algorithm with loop specific feedback to achieve target kinematics of the glenohumeral joint. Since the maximum force a muscle can produce was proportional to the pCSA, kinetic constraints of individual muscles were assigned from maximum voluntary contraction (MVC) (Table 1), as the product of muscle pCSA and specific strength^21–24^, whereby the specific strength was set to be 25 N/cm^26^.

**Table 1:** Prior loading ratios and kinetic constraints of the controlled muscles.

	Deltoid			ISP/TM	SSC	SSP
	MD	AD	PD			
Loading ratio	1	0.43	0.17	0.78	0.22	10°: 0.99 30°: 0.52 60°: 0.30
Physiological cross sectional area (cm^2^)	9.64	4.70	5.44	12.30	15.60	5.26
kinetic constraint (N)	241	254		308	390	132

*MD* Middle deltoid, *AD* Anterior deltoid, *PD* Posterior deltoid, *ISP/TM* Infraspinatus/Teres minor, *SSC* Subscapularis, *SSP* Supraspinatus.

Three modes of glenohumeral motion were implemented as follows:

#### Middle deltoid dominant abduction

The middle deltoid (MD) was configured to be the prime mover for abduction. The setpoint of the control loop was the target abduction angle, the process variable was the current abduction angle, and the control variable is the force applied by the simulated MD. A proportional–integral–derivative (PID) controller in the loop regulates the activation of MD force. During activation of the MD, SSP force is computed and applied according to the prior loaded muscle ratios, which is varied at different abduction levels (Table 1).

#### Anterior and posterior deltoid dominant flexion/extension

AD and PD are configured to be the prime movers for F/E. The setpoint of the control loop was target flexion/extension angle, the process variable was the current flexion/extension angle, and the control variables were the force applied by the simulated AD and PD forces. Two PID controllers in the loop tune the activation and the distribution of AD and PD forces.

#### Subscapularis and infraspinatus & teres minor dominant internal/external rotation

SSC and ISP/TM are set to be the prime movers for IR/ER. The setpoint of the control loop was target rotation angle, the process variable was the current rotation angle, and the control variables were the force applied by the simulated SSC and ISP/TM forces. Two PID controllers in the loop tune the activation and the distribution of SSC and ISP/TM forces.

Since the presence of muscle redundancy and co-activation of agonist–antagonist pairs, the three independent control loops work simultaneously, but asynchronously in disparate sequences for different motions as described below (Fig. 1b).

#### Cascade loading algorithm—active abduction

During active abduction with controlled F/E and IR/ER, the abduction control loop is determined to be the primary and the F/E and IR/ER loops are chosen to be the secondary control loops. The setpoint of the abduction angle is implemented by an activation controller, tuning the control variables of MD and SSP forces. Afterwards the control variables in the secondary loops, the total force applied to muscle couples AD&PD and SSC&ISP/TM, are calculated according the outputted MD force and prior muscle ratios. Subsequently the total forces are distributed to individual muscles by the outputs of the distribution controllers to maintain constant position in the secondary DOFs. In order to avoid muscle slackening the output range of the distribution controller is set to be 5–95% of the total force of antagonist muscles, so the individual muscle in the force couple is never unloaded.

#### Parallel loading algorithm—active flexion/extension and rotation

For active F/E or IR/ER with controlled abduction, MD no longer acts as the prime actuator to command muscles in other DOF and the sequence of three control loops changes to be parallel. The summations of two muscles couples in F/E and IR/ER are regulated individually by the activation controllers. For example, in active flexion with controlled elevation and rotation, activation and distribution controllers run synchronously to modulate the forces of AD and PD. The abduction and rotation loops run concurrently to maintain constant abduction and rotation.

The parameters for the controllers in each loop were tuned for two different forms of commands: targeted motion trajectories, and constant set angles in each DOF, respectively (e.g. abduction trajectory and constant abduction angle during flexion trajectory). The parameters were first tuned using the classical Ziegler-Nichols method, and then manually adjusted to optimize response time and reduce steady state error and overshoot (Table 2).

**Table 2:** Parameters of all PID controllers in the simulator.

Controller parameters			Proportional gain (Kc)	Integral time (Ti, min)	Derivative time (Td, min)
Abduction	Activation controller	Profile	2.600	0.023	0.000
		Constant	1.400	0.006	0.001
Flexion & extension	Distribution controller	Profile	0.030	0.050	0.000
		Constant	0.050	0.070	0.000
	Activation controller		0.030	0.050	0.000
Internal & external rotation	Distribution controller	Profile	0.030	0.060	0.000
		Constant	0.050	0.050	0.000
	Activation controller		0.020	0.050	0.000

### Specimen preparation and mounting

Three fresh frozen cadaveric shoulders (mean age: 59.3 ± 5.0; 3 males; 3 right) were obtained from a licensed human tissue facility for testing. The specimens had no history of glenohumeral osteoarthritis or cuff tear arthropathy. After thawing at room temperature for 12 h, the shoulders were transected at approximately 20 cm from proximal humerus, and all skin and subcutaneous tissue were resected. A Kirschner-wire, was pinned into distal humerus parallel to the transepicondylar axis of the elbow as a substitute for the medial and lateral epicondylar digitization. The inferior part of scapular was potted in a custom-made box using a three-component casting resin (Rencast FC 52/53, DT982, Gössl&Pfaff GmbH, Germany), and then mounted rigidly to the test rig in 10° forward inclination using four threaded rods to approximate its physiologic orientation on the thorax (Fig. 1a right). The distal humerus was cemented in a brass cylinder with the same resin and fixed with the forearm mass replacement (3 kg, 3.2% body weight). Two passive tracking tools were pinned directly into the humerus and scapula. Afterwards the deltoid was resected and a cortical screw was fixed at the deltoid insertion. Three low friction cables (Ultra cat 0.65 mm, Berkley, USA), representing three heads of the deltoid, were knotted to the screw and routed through three muscle guides, where two were attached to the supero-lateral border of the acromion and one to the lateral rim of the clavicle. To simulate rotator cuff muscles, the rotator cuff muscles were dissected from their respective fossae and three low friction cables were sutured to the musculotendinous junctions of the SSP, SSC and ISP/TM using the Krackow stitch, and passed through respective muscle guides and pulley sets along their lines of action (Fig. 1a). An initial force of 10–20N was applied to each muscle to center the humeral head in glenoid fosse and prevent dislocation. The resting abduction angle was approximately 10°.

### Simulations and data analysis

The specimens were moved passively through full range of motion several times and were kept moist to reduce the effect of viscoelasticity and thereby minimize hysteresis. Afterwards, different planar and non-planar motions of daily activities were performed as follows:

#### Planar motions

Active Abduction: 20°~60° abduction at 0° F/E and 0° IR/ER;

Active Flexion: 0°~30° flexion at 50° abduction and 0° IR/ER;

Active Extension: 0°~−30° extension at 50° abduction and 0° IR/ER;

Active Internal Rotation: 0°~45° IR at 30° abduction and 0° F/E;

Active External Rotation: 0° ~  − 45° ER at 30° abduction and 0° F/E.

#### Non-planar motion

Active Extension and Internal Rotation: 0° ~  − 30° extension simultaneously coupling with 0° ~ 30° IR at 40° abduction.

The target angular velocity of motion was set to 0.5°/s.

Each motion was executed 3 times in each specimen.

Kinematic data including angular rotations and translations in three DOF and kinetic data of the actual force of all simulated muscles were recorded. Data were analyzed in increments of 1°. The kinematic rotational accuracy of the simulator was computed as the mean deviation and root mean square error (RMSE) between the actual and desired joint angles. The kinematic repeatability and reliability was calculated as the maximum and average standard deviation (SD) of rotation angles and the intraclass correlation coefficient (ICC) of translations over the repeated motions. The reference position for translations was defined at 20° abduction, at which point the initial phase (10° ~ 20° abduction) of muscle loading has been completed. The kinetic reliability was measured as the ICC of individual muscle forces over repeated motions. ICC estimates and their 95% confident intervals (CI) were calculated using SPSS statistical package version 24 (SPSS Inc, Chicago, IL) based on a single-measurement, absolute-agreement, 2-way mixed-effects model. ICC less than 0.5 are indicative of poor reliability, values between 0.5 and 0.75 indicate moderate reliability, values between 0.75 and 0.9 indicate good reliability, and values greater than 0.90 indicate excellent reliability^27^ Generally, data is reported for all three specimens together. In one exception the control variables muscle force and glenohumeral translation, which is specimen specific, was reported individually for one specimen.

## Results

### Kinematic performance—joint angles and translation

**Figure 3:**
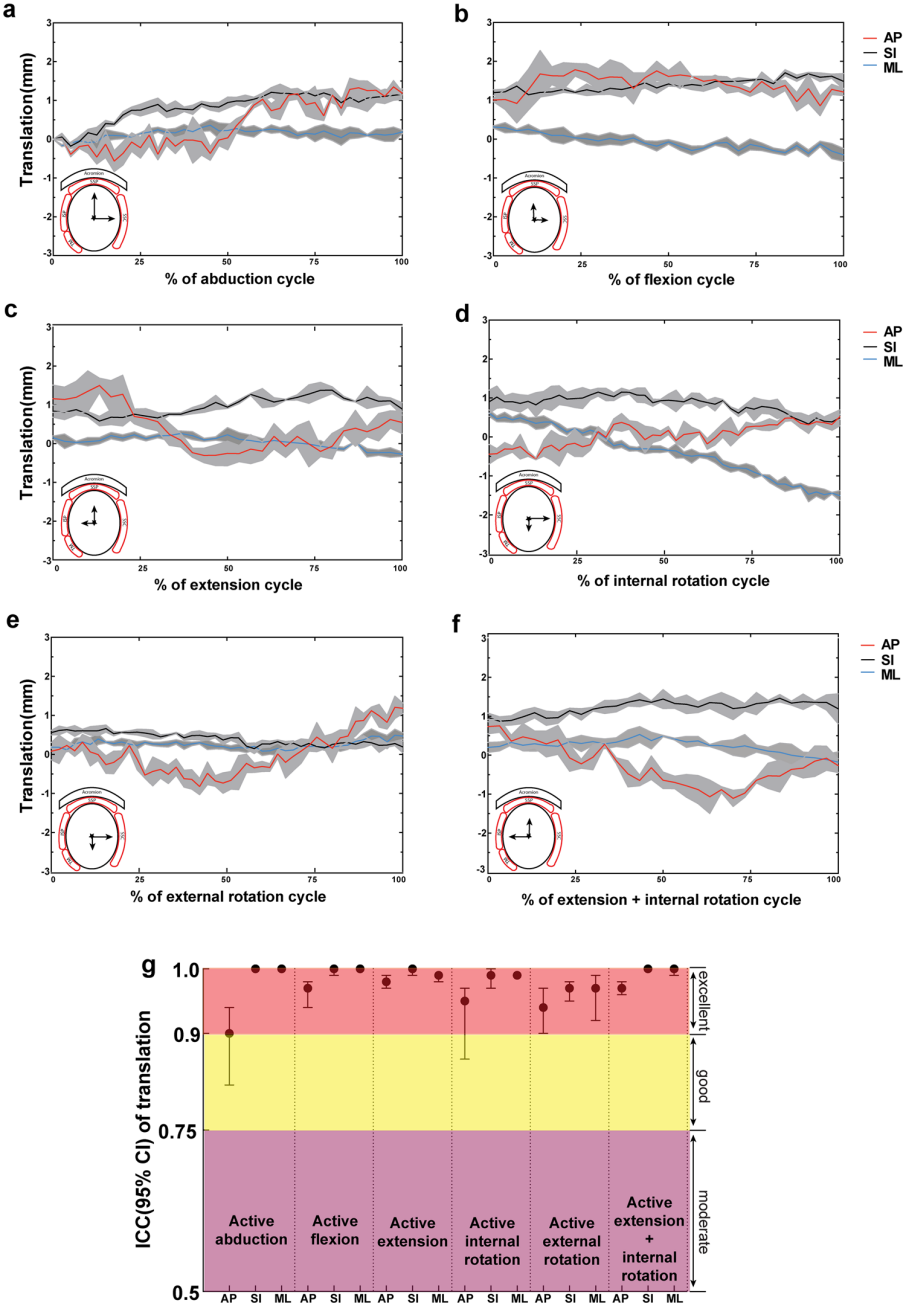
The glenohumeral translations (anterior/posterior, AP; superior/inferior, SI; medial/lateral, ML) during active abduction (**a**), flexion (**b**), extension (**c**), internal rotation (**d**), external rotation (**e**) and extension&internal rotation (**f**) cycles (Data from specimen 2). The grey areas represent standard deviations. The intraclass correlation coefficient (ICC) of AP, SI and ML translations over the repeated motions are delineated (**g**) (Data from all three specimens).

Good accuracy and repeatability were observed in all three rotational DOF investigated (Fig. 2). For *active abduction to 60°*, kinematic performance were excellent with a maximum mean deviation of 1.32°, RMSE of 0.75°, and maximum and average SDs of 1.01° and 0.29° for the deviation between the setpoint and process variable respectively (Table 3). For *active flexion and extension* of 30° to − 30°, maximum mean deviations and RMSEs were ≤ 1.74° and 0.89°, and maximum and average SDs were ≤ 1.02° and 0.51°, respectively. Similar performance was observed during *internal and external rotation*, with maximum mean deviations of 1.61° and 2.35°, RMSEs of 0.62° and 0.63°, maximum SDs of 0.76° and 0.82°, and average SDs of 0.39° and 0.41° (Table 3) respectively. The secondary 2 DOF during planar motions tracked well with a maximum mean deviation ≤ 0.80° during elevation, ≤ 1.18° during flexion/extension and ≤ 0.80° during rotation. Moreover, the measured translations of the humeral head in AP, SI, ML direction were highly reliable, with the 95% CIs of ICC estimates ≥ 0.90 in most instances (13/15) during active planar motions in all three specimens (Fig. 3).

**Table 3:** Parameters of all PID controllers in the simulator.

Simulations		Maximum mean deviation (°)	Root mean square error (°)	Maximum standard deviation (°)	Average standard deviation (°)
Active abduction (20–60°)	**ABD**	**1.32**	**0.75**	**1.01**	**0.29**
	F/E	0.80	0.26	0.33	0.18
	IR/ER	0.49	0.17	0.25	0.14
Active flexion (0–30°)	ABD	0.81	0.44	0.50	0.31
	**F/E**	**1.74**	**0.87**	**0.59**	**0.30**
	IR/ER	0.42	0.24	0.45	0.24
Active extension (0–30°)	ABD	1.18	0.46	0.68	0.32
	**F/E**	**1.58**	**0.89**	**1.02**	**0.51**
	IR/ER	0.44	0.24	0.35	0.21
Active internal rotation (0–45°)	ABD	0.55	0.19	0.21	0.11
	F/E	0.44	0.16	0.31	0.17
	**IR/ER**	**1.61**	**0.62**	**0.76**	**0.39**
Active external rotation (0–45°)	ABD	0.73	0.30	0.69	0.22
	F/E	0.80	0.36	0.90	0.27
	**IR/ER**	**2.35**	**0.63**	**0.82**	**0.41**
Active extension & internal rotation (0–30°)	ABD	0.82	0.36	0.67	0.37
	**F/E**	**1.64**	**1.13**	**1.21**	**0.67**
	**IR/ER**	**1.42**	**0.86**	**0.85**	**0.46**

*ABD* Abduction, *AP* Anterior–posterior, *F/E* Flexion/extension, IR/ER.

Significant values are in bold.

As to more complex non-planar motion of 0° ~  − 30° extension and coupled 0° ~ 30° internal rotation, comparable performance was observed to the purely planar motions; maximum mean deviations and maximum SDs were ≤ 1.64° and 1.21° for the target DOFs respectively (Table 3). The 95% CIs of ICC of three translational DOF were > 0.90.

### The kinetic performance—muscle force simulation

**Figure 4:**
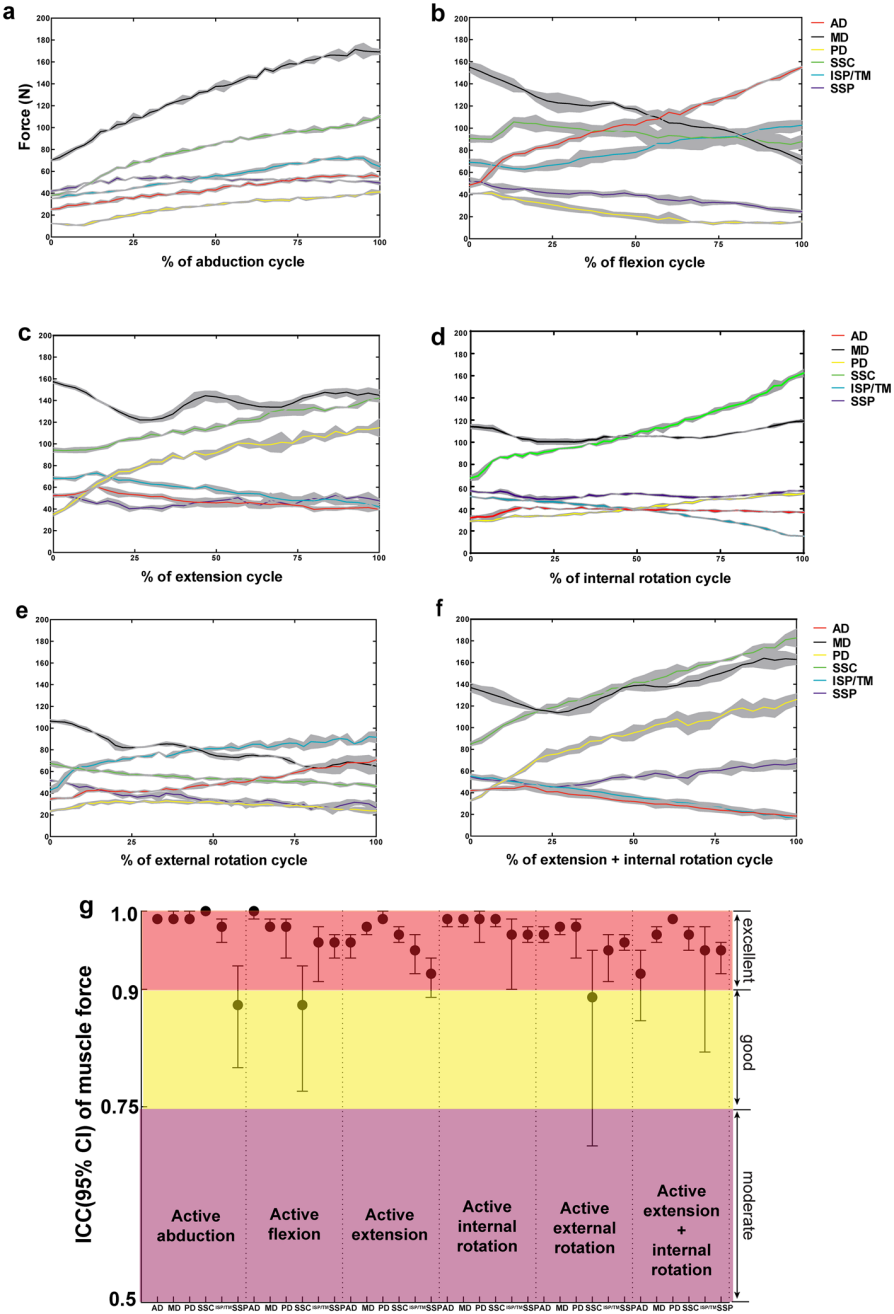
The actuated muscle forces (anterior deltoid, AD; middle deltoid, MD; posterior deltoid, PD; subscapularis, SSC, infraspinatus&teres minor, ISP/TM; supraspinatus, SSP) during active abduction (**a**), flexion (**b**), extension (**c**), internal rotation (**d**), external rotation (**e**) and extension&internal rotation (**f**) cycles (Data from specimen 2). The intraclass correlation coefficient (ICC) of AD, MD, PD, SSC, ISP/TM and SSP muscle forces over the repeated motions are delineated in (**g**) (Data from all three specimens).

For the muscle loads produced in one specimen (number 2) for active abduction, the prime mover “middle deltoid” had to produce an average force of 169.2 ± 2.3N at 60° (Fig. 4a). The force couples of AD&PD and SSC&ISP/TM were activated to maintain constant angles in secondary DOF. For active flexion and extension cycle, the prime mover AD&PD were highly actuated during the motion, with an average force of 155.0 ± 0.9N and 114.9 ± 7.9N at 30° flexion and extension, respectively (Fig. 4b and c). Likewise, SSC and ISP/TM drove internal/external rotation and the average force were 162.4 ± 3.5N and 91.6 ± 6.0N at the end of rotation cycle (Fig. 4d and e). For active extension coupled with internal rotation, the prime mover PD and SSC were highly actuated during motion, while antagonists AD and ISP/TM force was low (Fig. 4f).

The reliabilities of all individual simulated muscle forces actuated in the simulator were evaluated (Fig. 4g). During planar and non-planar motions, the 95% CIs of ICC estimates were > 0.90 for most instances (30/36), with a minimum ICC (95% CI) value of 0.88 (0.80–0.93) for SSP in active abduction.

## Discussion

A physiologic shoulder simulator with active simulated muscle actuation was designed and developed to replicate different planar and non-planar motions of daily activities. The capability, accuracy, repeatability and reliability were demonstrated and quantified under various planar and non-planar motions. The kinematic accuracy of maximum mean deviations and RMSE during all forms of motions was ≤ 2.35° and 1.13°, and repeatability of maximum and average SD was ≤ 1.21° and 0.67°, respectively, indicating effective tracking of target motions. The reliabilities of translational kinematics and kinetics were generally excellent with ICC of translations and simulated muscle forces ≥ 0.90 and 0.88, respectively.

One of the most remarkable characteristic of human shoulder is that it has the greatest range of motion of all the human diarthrodial joints, and possesses a delicate balance between mobility and stability^1^ Dynamic muscular control is thus of particular interest in shoulder biomechanics, and a physiologic loading pattern is an important prerequisite in many research questions during in vitro simulation. In previous studies, different sets of periarticular muscle loading ratios were calculated and applied to replicate the force pattern of physiologic muscle contractions: force simplified equal for all muscles, as well as various ratios deriving from physiological cross sectional area (pCSA) and electromyographic (EMG) activation data have found application^24,28–30^ No significant differences in resultant shoulder kinematics were found among different sets of physiologic muscle ratios^14^ In the active shoulder simulator presented in the current study, the two most important muscular groups (deltoid and rotator cuff) were simulated and designated as the prime mover dominating their respective primary DOF. The prescribed loading ratio obtained from pCSA and EMG data and three kinetic loops with multiple PID controllers constituted the core concept in the loading algorithm utilized. The operation sequences of individual loops were distinct to accommodate different types of movements under the kinetic constraints. On the basis of rational design, the excellent performance in resultant kinematics of this superior simulator indicated the capability of reproducing in vivo loading patterns and self-tuning to match the unique joint geometry and soft tissue condition of individual shoulder, which would provide reliable baseline for subsequent alterations, for instance to simulate injury or repair.

Regarding previous in vitro muscle-driven shoulder simulators in the literature, most of them were confined to perform pure abduction with constant muscle contraction velocity or linear increased muscle force in an open-loop strategy^14,15,31,32^ Thus the accuracy of following desired motion trajectory was not quantifiable. Wuelker et al. reported the repeatability of average SDs during elevation to be 0.80° for abduction, 0.75° for ante-/retroflexion and 1.36° for rotation^15^ Kedgley et al. reported the repeatability of maximum SDs to be less than 2° for both the plane of abduction and rotation^14^ Another advanced simulator presented by Giles et al. realized controlled planar motions in three DOF accurately with RMSE < 1°and average SD < 0.5^16^ Compared with aforementioned simulators, the kinematic performance of this novel simulator is similar to the best presented to date (from Giles et al.), with RMSE ≤ 1.13° and average SD ≤ 0.67°, even when performing complex non-planar motions. Nonetheless, Giles’s system was completely depended on gravity as the primary shoulder adductor and the deltoid muscle was configured to dictate the activation of other muscles. Its capability is limited when performing secondary DOF motions (e.g., plane of elevation and axial rotation) at low abduction levels. Furthermore, its reliability for producing glenohumeral translation and individual simulated muscle forces over repeated motions were not evaluated and it was not capable of performing complex non-planar motions in multiple DOF.

The average MD and SSP force of all specimens during repeated abduction in the current study was 176.0 ± 6.5N and 51.6 ± 2.1N at 60°. Similar force magnitudes were reported in the computer simulation models from Oizumi et al. and Van der Helm^33,34^ Besides, shoulder translation reported in previous active simulations in vitro ranged from 1 ~ 2 mm as a ball and socket behavior, to a gross translation of ~ 9–10mm^31,35,36^ Similar deviations in translation magnitude were observed for the three specimens simulated in this study (from 3 to 10 mm), illustrating specimen variability. Taken the redundant muscle actuation into consideration, multiple force distributions and different activation levels of agonist–antagonist pairs are possible to generate the same joint kinematics^21^ The glenohumeral translations are highly sensitive to the simulated muscle forces applied as well as to different forms of motion^1^ This is the cause for the difficulty in physiologic muscle simulation in vitro and comparing data from different test setups and subjects. Nevertheless, according to the accepted standard of ICC, the reliabilities of translations and muscle forces outcome in all simulated motions were generally excellent for this new simulator, illustrating the robustness and efficacy of the apparatus design.

Despite the conception of this active simulator has endeavored to recreate in vivo shoulder biomechanics, there are some limitations in the work. The primary one is the lack of the scapulothoracic motion simulation. The scapular moves across the thorax in vivo during arm elevation, with increased internal rotation, increased upward rotation and increased posterior tilting^32^ In previous simulators realizing scapulohumeral rhythm, motion was simplified as a two-dimensional linear relationship of the upward rotation of the scapula and the elevation of the arm^16,37^ Further work of our own has emphasized the implementation of scapulothoracic motion in all three DOF but will not be reported herein. A further limitation is the limited range of motion for flexion and extension. Since the remaining simulated muscles contributing to flexion/extension e.g. pectoralis major, biceps brachii, latissimus dorsi and teres major were not included in muscle simulation, large magnitude of flexion/extension are not achievable under the predeterminated kinetic constrains. Another limitation is the speed of our simulator with just 0,5 deg/s compared to the literature (Wuelker et al. ~ 3 deg /s, Kedgley et al. ~ 3,5 deg/s, Giles et al. ~ 1 to 4 deg/s). However, it should be noted that all of these angular motions can be seen as quasi-static in nature and do not represent fully dynamic motions such as in sports (i.e. baseball pitch). While there have been some efforts in this regard by other groups, it was not a goal of our current study^11,38^ A last limitation is the relatively simple representation of muscle. The classical methodology to represent muscles by single line of action was retained in this study may not suffice to mimic the moments produced by muscles with large attachment sites e.g. infraspinatus/teres minor. The lines of action of the muscle forces are respected, since the origin and insertions are replicated by the cable attachments to the specimen (insertion) and guides (origin). Slight changes in the force vector due to changes in muscle belly geometry during contraction, can however not be reproduced by this method. Developing a better approach for muscle simulation in vitro would be worth pursuing in further work.

## Conclusion

A new shoulder simulator with muscle simulation was built to reproduce physiological kinematics and kinetics in all DOF. The performance of the design was systematically evaluated in elaborate kinetic and kinematic outcome variables. This is the first model that was verified to perform all forms of planar and non-planar glenohumeral motions in an accurate and repeatable manner. It provides an important platform for investigating the biomechanics of physiological and pathological shoulder and as well as novel newly proposed surgical interventions.

## Data Availability

The authors confirm that the data supporting the findings of this study are available within the article.
